# Classifying microscopic images as acute lymphoblastic leukemia by Resnet ensemble model and Taguchi method

**DOI:** 10.1186/s12859-022-04558-5

**Published:** 2022-01-11

**Authors:** Yao-Mei Chen, Fu-I Chou, Wen-Hsien Ho, Jinn-Tsong Tsai

**Affiliations:** 1 School of Nursing, Kaohsiung Medical University, Kaohsiung, 807 Taiwan; 2 Superintendent Office, Kaohsiung Medical University Hospital, Kaohsiung, 807 Taiwan; 3 Department of Electrical Engineering, National Kaohsiung University of Science and Technology, Kaohsiung, 807 Taiwan; 4 Department of Healthcare Administration and Medical Informatics, Kaohsiung Medical University, Kaohsiung, 807 Taiwan; 5 Department of Medical Research, Kaohsiung Medical University Hospital, Kaohsiung, 807 Taiwan; 6 Department of Computer Science, National Pingtung University, Pingtung, 900 Taiwan

**Keywords:** Acute lymphoblastic leukemia, Microscopic image, ensemble model, Resnet model, Algorithm hyperparameter, Taguchi experimental method

## Abstract

**Background:**

Researchers have attempted to apply deep learning methods of artificial intelligence for rapidly and accurately detecting acute lymphoblastic leukemia (ALL) in microscopic images.

**Results:**

A Resnet101-9 ensemble model was developed for classifying ALL in microscopic images. The proposed Resnet101-9 ensemble model combined the use of the nine trained Resnet-101 models with a majority voting strategy. Each trained Resnet-101 model integrated the well-known pre-trained Resnet-101 model and its algorithm hyperparameters by using transfer learning method to classify ALL in microscopic images. The best combination of algorithm hyperparameters for the pre-trained Resnet-101 model was determined by Taguchi experimental method. The microscopic images used for training of the pre-trained Resnet-101 model and for performance tests of the trained Resnet-101 model were obtained from the C-NMC dataset. In experimental tests of performance, the Resnet101-9 ensemble model achieved an accuracy of 85.11% and an F_1_-score of 88.94 in classifying ALL in microscopic images. The accuracy of the Resnet101-9 ensemble model was superior to that of the nine trained Resnet-101 individual models. All other performance measures (i.e., precision, recall, and specificity) for the Resnet101-9 ensemble model exceeded those for the nine trained Resnet-101 individual models.

**Conclusion:**

Compared to the nine trained Resnet-101 individual models, the Resnet101-9 ensemble model had superior accuracy in classifying ALL in microscopic images obtained from the C-NMC dataset.

## Background

Acute lymphoblastic leukemia (ALL) is a cancer of the lymphoid line of blood cells characterized by development of numerous immature lymphocytes. As in acute leukemia, ALL progresses rapidly and is typically fatal within weeks or months if left untreated. If ALL is diagnosed in an early stage, however, curative treatment may be possible. Diagnosis is typically based on a complete blood count and microscope analysis of cell morphology, both of which are often performed manually by medical laboratory scientists. Although these tasks can be automated, the required equipment currently has a high cost and limited availability [1, 2]. An automated system that uses relatively low-cost and easily obtained microscopic images for diagnosis of leukemia would have many advantages. Therefore, artificial intelligence models for automatically detecting ALL in microscopic images are urgently needed.

### Literature review

Vogado et al. [3] extracted features from blood smear images by using pre-trained convolutional neural networks (CNNs) to obtain unique image descriptions. The authors evaluated several feature selection techniques and performed principal component analysis to select the features of the final descriptor. An ensemble model comprising support vector machine, multilayer perceptron, and random forest was then used to classify images as healthy or pathological. Rehman et al. [4] improved accuracy in diagnosing ALL by using a computer-aided system that integrated image processing and deep learning techniques. The authors proposed a method of classifying ALL in stained bone marrow images. Robust segmentation and deep learning techniques were used to train the CNN to classify bone marrow images accurately. Shafique and Tehsine [5] deployed a pre-trained AlexNet for automated detection and classification of ALL. The authors then classified ALL into subtypes L1, L2, and L3 in the French/American/British classification systems. The last layers of the pre-trained AlexNet were replaced with new layers for classifying input images into four classes: L1, L2, L3, and Normal. A data augmentation technique was also used to avoid overtraining. Liu and Long [1] proposed an ensemble model that used bagging ensemble learning method for training in ALL classification. The learning efficiency and classification accuracy of the proposed ensemble model was enhanced by using augmented images of ALL and elaborately designed training subsets for model training. In their preliminary test set, the proposed ensemble model obtained a weighted F_1_-score of 0.84. Prellberg and Kramer [2] presented a simple and effective classification approach that used a ResNeXt CNN with squeeze-and-excitation modules. Preliminary tests of their approach in the C-NMC-2019 dataset achieved an average weighted F_1_-score of 0.8789 in 24 training runs. Kassani et al. [6] presented a hybrid system for automated classification of leukemic B-lymphoblasts. The hybrid system integrated two CNNs (VGG16 and MobileNet) and transfer learning to extract features from input images of leukemic B-lymphoblasts. The proposed system fused features from selected intermediate layers to obtain an auxiliary feature set, which was used for further improvement of classification accuracy. Additionally, features extracted from lower levels were integrated in higher dimension feature maps, which not only improved the capability to discriminate intermediate features, but also avoided the problem of vanishing/exploding network gradients. Loey et al. [7] proposed two automated classification models for detecting leukemia in blood microscopic images. Use of transfer learning in the two models yielded several advantages over traditional approaches. Their first classification model pre-processed blood microscopic images then used AlexNet, a pre-trained deep CNN, for feature extraction. The AlexNet enabled application of numerous well-known classifiers. Their second classification model used pre-processed images to fine tune the AlexNet for both feature extraction and classification.

Notably, the above literature on detection of leukemia in microscopic images reveal that most related studies performed so far have investigated individual models for classifying microscopic images of ALL. Using an ensemble model, in which classification is based on the majority results, can reduce image classification errors. Moreover, few studies have discussed how algorithm hyperparameters affect classification accuracy in a pre-trained CNN model. Therefore, the motivations for this study were the lack of research on an ensemble model and lack of research on effect of algorithm hyperparameters on accuracy of a pre-trained CNN model.

### Objectives

This study had two objectives. The first objective was to determine the best combination of algorithm hyperparameters for the pre-trained Resnet-101 model. The second objective of this study was to establish an ensemble model that used multiple trained Resnet-101 models and a majority voting strategy to classify ALL in microscopic images. The method of integrating an ensemble model and a majority voting strategy can solve the problem that different single models classify the same image with different symptoms. That is, classification of images by the ensemble model is analogous to classification of images according to the consensus of medical laboratory scientists. In a pre-trained Resnet-101 model, learning speed and classification quality are determined by algorithm hyperparameters that are set before the learning process begins. In subsequent training, however, a pre-trained Resnet-101 model may require different algorithm hyperparameters (e.g., optimizer, learning rate, and mini-batch size) to improve its classification accuracy. This study used Taguchi method, which is a systematic and robust experimental method, to generate the best combination of algorithm hyperparameters for a pre-trained Resnet-101 model. In experimental comparisons, the Resnet101 ensemble model had superior classification accuracy compared to trained Resnet-101 individual models and had excellent accuracy in classifying ALL in microscopic images.

### Problem description

Acute lymphoblastic leukemia, a cancer type that affects the blood and bone marrow, is characterized by overproduction of immature white blood cells, called lymphoblasts or leukemic blasts. Since the bone marrow cannot produce adequate numbers of red cells, normal white cells, and platelets, people with ALL are susceptible to anemia and recurrent infections as well as easy bruising and bleeding. As a result, blast cells that spill out of the bone marrow and into the bloodstream can accumulate in various organs, including the lymph nodes (glands), spleen, liver, and central nervous system (brain and spinal cord) [1, 2].

**Fig. 1:**
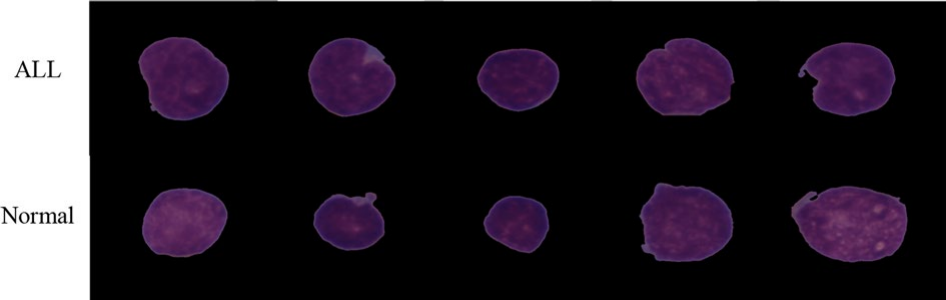
Representative microscopic images of ALL cells and Normal cells

Acute lymphoblastic leukemia occurs in approximately 25% of all pediatric cancers. When viewed under a microscope, immature leukemic blasts and normal cells are difficult to distinguish due to their similar morphology [8]. Figure 1 compares representative microscopic images of ALL cells and Normal cells.

Detecting ALL often requires review of numerous and highly similar microscopic images by a medical laboratory scientist, which can be extremely time consuming and burdensome. Additionally, medical personnel in rural and/or under-developed areas may lack adequate training in detecting ALL in blood microscopic images. Therefore, the considered problem was how to screen numerous highly similar blood microscopic images for ALL efficiently and accurately. To aid medical laboratory personnel in identifying ALL in blood microscopic images, an artificial intelligence model trained by deep learning method may be a useful tool.

## Results

The proposed Resnet101 ensemble model integrated multiple trained Resnet-101 models with a majority voting strategy for classifying ALL in microscopic images. The well-known pre-trained Resnet-101 model with its appropriate algorithm hyperparameters was trained to classify ALL in microscopic images. The training set of microscopic images used to train the pre-trained Resnet-101 model was obtained from the C-NMC dataset. The preliminary test set of microscopic images was used for performance evaluation of the trained Resnet-101 model. The experimental environment was Matlab R2019 and its toolboxes developed by MathWorks.

The experimental data used to test performance in classifying ALL in microscopic images included the training set and the preliminary test set. Table 1 shows the number of microscopic images in the training set and in the preliminary test set. To maintain compatibility with the CNN-based architecture and the developed software, each microscopic image was processed as a 224 × 224 × 3 image, where 3 is the number of color channels.

**Table 1:** Number of images in datasets for training and preliminary testing of performance in classifying ALL in microscopic images

Class	Training set	Preliminary test set	Total images
ALL	7272	1219	8491
Normal	3389	648	4037
Total images	10,661	1867	12,528

A pre-trained Resnet-101 model was selected for training process, and then tried to set different algorithm hyperparameters before the learning process began. The algorithm hyperparameters for the pre-trained Resnet-101 model in this study were ‘Optimizer’, ‘MiniBatchSize’, ‘MaxEpochs’, and ‘InitialLearnRate’. Optimizer was the training option. MiniBatchSize was a mini-batch at each iteration. MaxEpochs was the maximum number of training epochs. InitialLearnRate was an option for decreasing the learning rate during training.

A three-level OA of the minimum number of experiments for four factors is *L*_9_(3^4^). Table 2 shows the three-level *L*_9_(3^4^) OA, and Table 3 shows the factors and levels. The three levels for the ‘Optimizer’ hyperparameter (factor A) were ‘adam (adaptive moment estimation)’, ‘sgdm (stochastic gradient descent with a momentum)’, and ‘adam’. The three levels for the ‘MiniBatchSize’ hyperparameter (factor B) were 60, 65, and 70. The three levels for the ‘MaxEpochs’ hyperparameter (factor C) were 8, 10, and 12. The three levels for ‘InitialLearnRate’ hyperparameter (factor D) were 10^−4^, 10^−5^, and 10^−6^. Instead of 81 (3^4^) experiments, the *L*_9_(3^4^) OA required only 9 experiments.

**Table 2:** Three-level *L*_9_(3^4^) OA

Number of experiments	Factors			
	A	B	C	D
1	1	1	1	1
2	1	2	2	2
3	1	3	3	3
4	2	1	2	3
5	2	2	3	1
6	2	3	1	2
7	3	1	3	2
8	3	2	1	3
9	3	3	2	1

**Table 3:** Factors and levels

Factor (Algorithm hyperparameter)	Levels		
	1	2	3
A: Optimizer	adam	sgdm	adam
B: MiniBatchSize	60	65	70
C: MaxEpochs	8	10	12
D: InitialLearnRate	10^−4^	10^−5^	10^−6^

Table 4 shows the combinations of the four algorithm hyperparameters that combined the values in Tables 2 and 3 and were used in a pre-trained Resnet-101 model for classifying ALL in microscopic images.

**Table 4:** Combinations of four algorithm hyperparameters for a pre-trained CNN model

Number of experiments	Algorithm hyperparameters			
	Optimizer	MiniBatchSize	MaxEpochs	InitialLearnRate
1	adam	60	8	10^−4^
2	adam	65	10	10^−5^
3	adam	70	12	10^−6^
4	sgdm	60	10	10^−6^
5	sgdm	65	12	10^−4^
6	sgdm	70	8	10^−5^
7	adam	60	12	10^−5^
8	adam	65	8	10^−6^
9	adam	70	10	10^−4^

The algorithm hyperparameter combinations in Table 4 were used in three independent experimental runs in the training set of the pre-trained Resnet-101 model and in the preliminary test set of the trained Resnet-101 model. In tests of performance in classifying ALL in microscopic images, Table 5 shows the accuracy obtained in a single run and the average accuracy, standard deviation (SD), and *η* value obtained in three runs.

**Table 5:** Accuracy of the trained Resnet-101 model in classifying ALL in microscopic images when the algorithm hyperparameter combinations in Table 4 were used in three independent experimental runs

Experiments 1–9	Dataset	Runs of experiment			Average accuracy	SD	*η* value
		1	2	3			
1	Training set	0.9777	0.9796	0.9792	0.9788	0.0010	33.4870
	Preliminary test set	0.8045	0.8066	0.7927	0.8013	0.0075	14.0346
2	Training set	0.985	0.9864	0.9872	0.9862	0.0011	37.2024
	Preliminary test set	0.7916	0.7943	0.805	0.7970	0.0071	13.8487
3	Training set	0.9211	0.9218	0.9216	0.9215	0.0004	22.1026
	Preliminary test set	0.7477	0.7483	0.7483	0.7481	0.0003	11.9754
4	Training set	0.7892	0.7888	0.7893	0.7891	0.0003	13.5185
	Preliminary test set	0.6508	0.6508	0.6508	0.6508	0.0000	9.1385
5	Training set	0.9533	0.9538	0.9535	0.9535	0.0003	26.6572
	Preliminary test set	0.7783	0.7809	0.7788	0.7793	0.0014	13.1253
6	Training set	0.864	0.8639	0.8647	0.8642	0.0004	17.3420
	Preliminary test set	0.6909	0.6904	0.6888	0.6900	0.0011	10.1737
7	Training set	0.985	0.9877	0.985	0.9859	0.0016	37.0156
	Preliminary test set	0.8056	0.8013	0.7965	0.8011	0.0046	14.0288
8	Training set	0.9056	0.9057	0.9064	0.9059	0.0004	20.5282
	Preliminary test set	0.7268	0.7327	0.7338	0.7311	0.0038	11.4082
9	Training set	0.9796	0.9831	0.9831	0.9819	0.0020	34.8624
	Preliminary test set	0.7954	0.7868	0.7563	0.7795	0.0205	13.1318

**Fig. 2:**
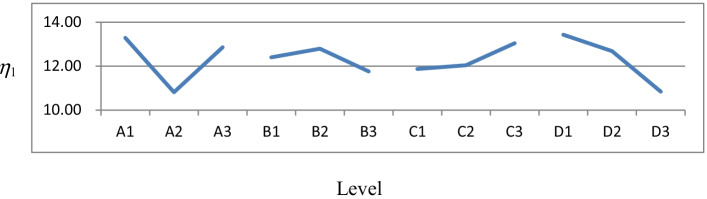
Plots of factor effects

Table 6 is the response table for each factor, and Fig. 2 plots the effects of the factors, which were obtained by computing the *η* value for each factor level in Table 5. Table 6 shows that factor levels 1, 2, 3, and 1 were selected for factors A, B, C, and D, respectively. Thus, the best factor-level combination was A1: adam, B2: 65, C3: 12, and D1: 10^−4^.

**Table 6:** Response table for each factor

Level	Factors			
	A	B	C	D
1	13.2862	12.4006	11.8722	13.4306
2	10.8125	12.7940	12.0397	12.6837
3	12.8563	11.7603	13.0432	10.8407
Effect	2.4737	1.0337	1.1710	2.5898
Maximum	13.2862	12.7940	13.0432	13.4306
Best level number	1	2	3	1
Best level value	adam	65	12	10^−4^

In the confirmation experiment, the best combination of algorithm hyperparameters (i.e., A1: adam, B2: 65, C3: 12, and D1: 10^−4^) was used in nine independent experimental runs of the trained Resnet-101 model, and the nine models were generated, namely Resnet-101-8249(#1), Resnet-101-8184(#2), Resnet-101-8452(#3), Resnet-101-8125(#4), Resnet-101-8061(#5), Resnet-101-8281(#6), Resnet-101-8307(#7), Resnet-101-8002(#8), and Resnet-101-8216(#9). For classifying ALL in microscopic images, Table 7 shows the accuracy achieved in the nine models for the training set and the preliminary test set. Table 7 also shows that the average accuracy and *η* value obtained in the nine models performed in the preliminary test set were 0.8209 and 14.9359, respectively, which exceeded those in each *L*_9_(3^4^) OA experiment (Table 5) in the preliminary test set. The best combination of algorithm hyperparameters in the response table can obtain the best result even though not all factor-level combinations were tested. Therefore, the best combination of algorithm hyperparameters obtained in the confirmation experiments was used in the trained Resnet-101 model for classifying ALL in microscopic images.

**Table 7:** Accuracy of the nine trained Resnet-101 individual models in classifying ALL in microscopic images when the best combination of hyperparameters was used in nine independent experimental runs

Model	Accuracy for the training set	Accuracy for the preliminary test set
Resnet-101-8249(#1)	0.9881	0.8249
Resnet-101-8184(#2)	0.9856	0.8184
Resnet-101-8452(#3)	0.9872	0.8452
Resnet-101-8125(#4)	0.9893	0.8125
Resnet-101-8061(#5)	0.9841	0.8061
Resnet-101-8281(#6)	0.9848	0.8281
Resnet-101-8307(#7)	0.9811	0.8307
Resnet-101-8002(#8)	0.9877	0.8002
Resnet-101-8216(#9)	0.9859	0.8216
Average accuracy	0.9860	0.8209
SD	0.0025	0.0136
*η* value	37.0637	14.9359

Additionally, ANOVA was performed to determine what factors had the greatest influence on the accuracy of the trained Resnet-101 model in classifying ALL in microscopic images. Table 8 shows the ANOVA results. Factors A (optimizer) and D (initialLearnRate) had the largest effects on accuracy. The percentage contributions of Factors A and D to experimental variance were 41.62% and 42.34%, respectively, and their total percentage contribution to experimental variance was 83.96%. Therefore, statistically significant factors in the accuracy of the trained Resnet-101 model in classifying ALL in microscopic images were A1 (adam) and D1 (10^−4^).

**Table 8:** Summary of ANOVA results

Factor	Sum of squares	Degrees of freedom	Variance	Expected sum of squares	Percentage contribution (%)
A: optimizer	10.4812	2	5.2406	10.4812	41.62
B: miniBatchSize	1.6333	2	0.8167	1.6333	6.49
C: maxEpochs	2.4063	2	1.2031	2.4063	9.56
D: initialLearnRate	10.6617	2	5.3309	10.6617	42.34
Error	0.0000	0			
*S*_*T*_	25.1825	8			100

Table 7 shows the high classification accuracy achieved by each model in the preliminary test set. In all models, classification accuracy in the preliminary test set exceeded that of all *L*_9_(3^4^) OA experiments (Table 5). Therefore, the nine trained Resnet-101 individual models were selected for inclusion in the ensemble model. The Resnet101 ensemble models comprised four ensemble models (Resnet101-3, Resnet101-5, Resnet101-7, and Resnet101-9). A microscopic image classified as ALL cell by most models was considered an ALL class, and a microscopic image classified as Normal cell by most models was considered a Normal class. The accuracy metric was used to compare performance in the trained Resnet-101 and Resnet101 ensemble models. Precision, recall, specificity, and F_1_-score were further used to validate classification performance. The results were depicted by creating a confusion matrix of actual class versus predicted class for the respective classes.

The comparison of classification accuracy in the four ensemble models in the preliminary test set were as follows.

The Resnet101-3 ensemble model, which combined Resnet-101-8249, Resnet-101-8184, and Resnet-101-8452 models, classified ALL in microscopic images by aggregating the results of a majority voting strategy. Table 9 shows the confusion matrices used to compare ALL and Normal classes in the Resnet-101-8249, Resnet-101-8184, Resnet-101-8452, and Resnet101-3 ensemble models for the preliminary test set.

**Table 9:** Confusion matrix for classification of images as ALL or Normal classes by the trained Resnet-101 individual models and by the Resnet101-3 ensemble model for the preliminary test set

Model			Actual classes	
			ALL	Normal
Resnet-101-8249	Predicted classes	ALL	1095	203
		Normal	124	445
Resnet-101-8184	Predicted classes	ALL	1047	167
		Normal	172	481
Resnet-101-8452	Predicted classes	ALL	1114	184
		Normal	105	464
Resnet101-3 ensemble	Predicted classes	ALL	1104	182
		Normal	115	466

Based on the data in Table 9, Table 10 shows the classifier accuracy, precision, recall, specificity, and F_1_-score obtained by the trained Resnet-101 individual models and by the Resnet101-3 ensemble model. When the preliminary test set was used, the accuracy of the Resnet101-3 ensemble model (0.8409) was higher than those of the Resnet-101-8249 and Resnet-101-8184 models but lower than that of the Resnet-101-8452 model. Other performance measures (i.e., precision, recall, specificity, and F_1_-score) for the Resnet101-3 ensemble model approximated those in the trained Resnet-101 individual models.

**Table 10:** Classification accuracy, precision, recall, specificity, and F_1_-score obtained by trained Resnet-101 individual models and by the Resnet101-3 ensemble model for the preliminary test set

Model	Accuracy	Precision	Recall	Specificity	F_1_-score
Resnet-101-8249	0.8249	0.8436	0.8983	0.6867	0.8701
Resnet-101-8184	0.8184	0.8624	0.8589	0.7423	0.8607
Resnet-101-8452	0.8452	0.8582	0.9139	0.7160	0.8852
Resnet101-3 ensemble	0.8409	0.8585	0.9057	0.7191	0.8814

The Resnet101-5 ensemble model applied a majority voting strategy to classify ALL in microscopic images. That is, the Resnet101-5 ensemble model classified ALL by aggregating the results of five models: Resnet-101-8249, Resnet-101-8184, Resnet-101-8452, Resnet-101-8125, and Resnet-101-8061 models. Table 11 shows the confusion matrices used for comparisons of ALL and Normal classes in the Resnet-101-8249, Resnet-101-8184, Resnet-101-8452, Resnet-101-8125, Resnet-101-8061, and Resnet101-5 ensemble models for the preliminary test set.

**Table 11:** Confusion matrix for classification of images as ALL and Normal classes by the trained Resnet-101 individual models and by the Resnet101-5 ensemble model for the preliminary test set

Model			Actual classes	
			ALL	Normal
Resnet-101-8249	Predicted classes	ALL	1095	203
		Normal	124	445
Resnet-101-8184	Predicted classes	ALL	1047	167
		Normal	172	481
Resnet-101-8452	Predicted classes	ALL	1114	184
		Normal	105	464
Resnet-101-8125	Predicted classes	ALL	1078	209
		Normal	141	439
Resnet-101-8061	Predicted classes	ALL	1030	173
		Normal	189	475
Resnet101-5 ensemble	Predicted classes	ALL	1100	173
		Normal	119	475

Based on the data in Table 11, Table 12 shows the classifier accuracy, precision, recall, specificity, and F_1_-score obtained by the trained Resnet-101 individual models and by the Resnet101-5 ensemble model. In the preliminary test set, the accuracy of the Resnet101-5 ensemble model (0.8436) was superior to that of all trained Resnet-101 individual models except Resnet-101-8452 model. Other performance measures (i.e., precision, recall, specificity, and F_1_-score) obtained by the Resnet101-5 ensemble model were not consistently superior or inferior to those of the trained Resnet-101 individual models.

**Table 12:** Classification accuracy, precision, recall, specificity, and F_1_-score obtained by trained Resnet-101 individual models and by the Resnet101-5 ensemble model for the preliminary test set

Model	Accuracy	Precision	Recall	Specificity	F_1_-score
Resnet-101-8249	0.8249	0.8436	0.8983	0.6867	0.8701
Resnet-101-8184	0.8184	0.8624	0.8589	0.7423	0.8607
Resnet-101-8452	0.8452	0.8582	0.9139	0.716	0.8852
Resnet-101-8125	0.8125	0.8376	0.8843	0.6775	0.8603
Resnet-101-8061	0.8061	0.8562	0.845	0.733	0.8505
Resnet101-5 ensemble	0.8436	0.8641	0.9024	0.733	0.8828

As in the Resnet101-7 ensemble model, the Resnet101-7 ensemble model used a majority voting strategy to classify ALL in microscopic images. However, the Resnet-7 ensemble model integrated seven models: Resnet-101-8249, Resnet-101-8184, Resnet-101-8452, Resnet-101-8125, Resnet-101-8061, Resnet-101-8281, and Resnet-101-8307 models. The Resnet101-7 ensemble model classified ALL by aggregating the results of the majority voting strategy. Table 13 shows the confusion matrices used to compare performance in classifying images in the preliminary test set as ALL or Normal classes in the seven trained Resnet-101 individual models and in the Resnet101-7 ensemble model.

**Table 13:** Confusion matrix for classification of images as ALL and Normal classes by the trained Resnet-101 individual models and by the Resnet101-7 ensemble model for the preliminary test set

Model			True classes	
			ALL	Normal
Resnet-101-8249	Predicted classes	ALL	1095	203
		Normal	124	445
Resnet-101-8184	Predicted classes	ALL	1047	167
		Normal	172	481
Resnet-101-8452	Predicted classes	ALL	1114	184
		Normal	105	464
Resnet-101-8125	Predicted classes	ALL	1078	209
		Normal	141	439
Resnet-101-8061	Predicted classes	ALL	1030	173
		Normal	189	475
Resnet-101-8281	Predicted classes	ALL	1114	216
		Normal	105	432
Resnet-101-8307	Predicted classes	ALL	1090	187
		Normal	129	461
Resnet101-7 ensemble	Predicted classes	ALL	1116	176
		Normal	103	472

Based on the data in Table 13, Table 14 shows the classification accuracy, precision, recall, specificity, and F_1_-score obtained by the trained Resnet-101 individual models and by the Resnet101-7 ensemble model. In the preliminary test set, the Resnet101-7 ensemble model achieved an accuracy of 0.8506, which was superior to those of the trained Resnet-101 individual models. All other performance measures (i.e., precision, recall, specificity, and F_1_-score) obtained for the Resnet101-7 ensemble model were higher than those for the trained Resnet-101 individual models. That is, the Resnet101-7 ensemble model had superior accuracy in classifying ALL in microscopic images.

**Table 14:** Classification accuracy, precision, recall, specificity, and F_1_-score obtained by trained Resnet-101 individual models and by the Resnet101-7 ensemble model for the preliminary test set

Model	Accuracy	Precision	Recall	Specificity	F_1_-score
Resnet-101-8249	0.8249	0.8436	0.8983	0.6867	0.8701
Resnet-101-8184	0.8184	0.8624	0.8589	0.7423	0.8607
Resnet-101-8452	0.8452	0.8582	0.9139	0.716	0.8852
Resnet-101-8125	0.8125	0.8376	0.8843	0.6775	0.8603
Resnet-101-8061	0.8061	0.8562	0.845	0.733	0.8505
Resnet-101-8281	0.8281	0.8376	0.9139	0.6667	0.8741
Resnet-101-8307	0.8307	0.8536	0.8942	0.7114	0.8734
Resnet101-7 ensemble	0.8506	0.8638	0.9155	0.7284	0.8889

The Resnet101-9 ensemble model, which combined Resnet-101-8249, Resnet-101-8184, Resnet-101-8452, Resnet-101-8125, Resnet-101-8061, Resnet-101-8281, Resnet-101-8307, Resnet-101-8002, and Resnet-101-8216 models, accurately classified ALL in microscopic images by using a majority voting strategy to aggregate the results of these nine models. Table 15 shows the confusion matrices used for comparisons of ALL and Normal classes. Table 15 is the confusion matrix for the classification performance of the nine trained Resnet-101 individual models and the Resnet101-9 ensemble model for the preliminary test set.

**Table 15:** Confusion matrix for performance of the trained Resnet-101 individual models and the Resnet101-9 ensemble model in classifying images in the preliminary test set as ALL or Normal classes

Model			Actual classes	
			ALL	Normal
Resnet-101-8249	Predicted classes	ALL	1095	203
		Normal	124	445
Resnet-101-8184	Predicted classes	ALL	1047	167
		Normal	172	481
Resnet-101-8452	Predicted classes	ALL	1114	184
		Normal	105	464
Resnet-101-8125	Predicted classes	ALL	1078	209
		Normal	141	439
Resnet-101-8061	Predicted classes	ALL	1030	173
		Normal	189	475
Resnet-101-8281	Predicted classes	ALL	1114	216
		Normal	105	432
Resnet-101-8307	Predicted classes	ALL	1090	187
		Normal	129	461
Resnet-101-8002	Predicted classes	ALL	1032	186
		Normal	187	462
Resnet-101-8216	Predicted classes	ALL	1099	213
		Normal	120	435
Resnet101-9 ensemble	Predicted classes	ALL	1118	177
		Normal	101	471

Based on the data in Table 15, Table 16 shows the classifier accuracy, precision, recall, specificity, and F_1_-score obtained by the trained Resnet-101 individual models and by the Resnet101-9 ensemble model. When the Resnet101-9 ensemble model was used in the preliminary test set, the accuracy was 0.8511, which was superior to the accuracies obtained by the trained Resnet-101 individual models. Other performance measures (i.e., precision, recall, specificity, and F_1_-score) obtained by the Resnet101-9 ensemble model were higher than those obtained by the trained Resnet-101 individual models. That is, the Resnet101-9 ensemble model had superior accuracy in classifying ALL in microscopic images.

**Table 16:** Classification accuracy, precision, recall, specificity, and F_1_-score obtained by the trained Resnet-101 individual models and by the Resnet101-9 ensemble model for the preliminary test set

Model	Accuracy	Precision	Recall	Specificity	F_1_-score
Resnet-101-8249	0.8249	0.8436	0.8983	0.6867	0.8701
Resnet-101-8184	0.8184	0.8624	0.8589	0.7423	0.8607
Resnet-101-8452	0.8452	0.8582	0.9139	0.716	0.8852
Resnet-101-8125	0.8125	0.8376	0.8843	0.6775	0.8603
Resnet-101-8061	0.8061	0.8562	0.845	0.733	0.8505
Resnet-101-8281	0.8281	0.8376	0.9139	0.6667	0.8741
Resnet-101-8307	0.8307	0.8536	0.8942	0.7114	0.8734
Resnet-101-8002	0.8002	0.8473	0.8466	0.7130	0.8469
Resnet-101-8216	0.8216	0.8377	0.9016	0.6713	0.8684
Resnet101-9 ensemble	0.8511	0.8633	0.9171	0.7269	0.8894

The classification results from Resent101-3, -5, -7, -9 ensemble models showed that the classification accuracy of multiple integrated models was higher than that of a single model and fewer integrated models. In this study, the ensemble models that integrated the largest number of models (i.e., nine models) had the highest classification accuracy. Therefore, the Resnet101-9 ensemble model combined with a majority voting strategy was used to classify ALL in microscopic images.

## Discussion

The preliminary test set contained 1867 microscopic images, including 1219 images of ALL cells and 648 images of Normal cells. Table 17 shows the numbers of images that the Resnet101-9 ensemble model classified incorrectly. 101 microscopic images were ALL cells but were incorrectly classified as Normal cells, and 36 images were incorrectly classified by nine individual models and should be reviewed by medical laboratory scientists. 177 microscopic images were Normal cells but were incorrectly classified as ALL cells, and 67 images were incorrectly classified by nine individual models and should be reviewed by medical laboratory scientists. Microscopic images of acute lymphoblastic leukemia were obtained from the C-NMC dataset for testing the performance of the proposed artificial intelligence methods. Classification accuracy of the preliminary test set in the previous studies [1, 2, 9] and the Resnet101-9 ensemble model is no more than 0.9. The authors coming from Medical University believed that some images from the C-NMC dataset were incorrectly labeled and needed further confirmation by medical laboratory scientists. In most object detection and classification problems encountered in the medical field, professional knowledge or experience is needed to label objects correctly. Therefore, a reliable dataset of correctly labeled objects is essential for model training and testing.

**Table 17:** Image classification errors by the Resnet101-9 ensemble model for the preliminary test set

Classification error status	Number of incorrect classifications	Numbers of microscopic images	Amount of incorrect classifications
	5	165, 261, 279, 355, 368, 388, 533, 570, 574, 632, 690, 857, 1010, 1095, 1235, 1254, 1301, 1355, 1522, 1606, 1625, 1682, 1709, 1715	24
	6	294, 377, 528, 544, 908, 912, 1099, 1219, 1408, 1433	10
ALL incorrectly classified as	7	210, 447, 525, 629, 646, 767, 799, 805, 855, 882, 887, 913, 1132, 1223, 1405, 1861	16
Normal	8	250, 389, 433, 612, 746, 976, 1031, 1127, 1277, 1361, 1492, 1515, 1521, 1652, 1692	15
	9	47, 179, 204, 219, 239, 295, 336, 427, 634, 692, 719, 737, 768, 843, 850, 859, 869, 910, 961, 1019, 1081, 1116, 1121, 1310, 1337, 1397, 1418, 1434, 1528, 1531, 1580, 1588, 1592, 1769, 1796, 1834	36
	5	60, 63, 67, 90, 127, 187, 240, 391, 431, 461, 465, 567, 787, 891, 946, 1335, 1365, 1367, 1441, 1449, 1485, 1487, 1514, 1538, 1634, 1723, 1739, 1758, 1823, 1865	30
	6	158, 173, 233, 258, 305, 313, 376, 405, 442, 464, 728, 747, 814, 866, 872, 933, 1062, 1074, 1123, 1149, 1275, 1591, 1603, 1629, 1696, 1729, 1787	27
Normal incorrectly classified as	7	236, 251, 298, 382, 446, 475, 516, 693, 698, 724, 898, 1111, 1126, 1175, 1195, 1265, 1295, 1377, 1399, 1431, 1473, 1530, 1716, 1815	24
ALL	8	13, 172, 220, 289, 420, 435, 484, 529, 627, 684, 775, 831, 949, 1063, 1119, 1247, 1263, 1379, 1411, 1537, 1545, 1590, 1624, 1673, 1732, 1759, 1820, 1840, 1850	29
	9	26, 35, 50, 54, 117, 142, 160, 171, 212, 214, 256, 259, 264, 299, 320, 340, 369, 421, 423, 469, 472, 530, 531, 536, 609, 643, 654, 682, 735, 786, 791, 840, 854, 864, 867, 896, 924, 930, 931, 963, 974, 980, 996, 1017, 1072, 1191, 1220, 1222, 1249, 1252, 1267, 1307, 1324, 1409, 1422, 1440, 1458, 1460, 1525, 1526, 1623, 1724, 1741, 1749, 1773, 1786, 1814	67

Number of incorrect classifications: The number of incorrect classifications of an image by the nine individual models

This study found that an appropriate combination of algorithm hyperparameter settings for a pre-trained Resnet-101 model is essential for accurately classifying ALL in microscopic images. In the trained Resnet-101 model, the best combination was Optimizer of ‘adam’, MiniBatchSize of 65, MaxEpochs of 12, and InitialLearnRate of 10^−4^. The results of this study indicate that a poor combination of algorithm hyperparameters for a pre-trained Resnet-101 model cannot accurately classify ALL in microscopic images. Although different trained Resnet-101 individual models have different accuracy in classifying ALL in microscopic images, the Resnet101 ensemble model used a voting mechanism to aggregate the classification results. That is, classification of microscopic images of ALL by the ensemble model is analogous to classification of microscopic images of ALL according to the consensus of medical laboratory scientists.

Although studies by Liu and Long [1], by Prellberg and Kramer [2], and by Mondal et al. [9] used the same database (C-NMC dataset), they used different image size and processing, different performance criteria, and did not provide confusion matrices. Therefore, prediction performance comparisons with these earlier studies are not possible. Liu and Long [1] reported a weighted F_1_-score of 0.84, weighted precision of 0.84, and weighted recall of 0.85 for their preliminary test set. Prellberg and Kramer [2] reported an average weighted F_1_-score of 0.8789, an average weighted precision of 0.8791, and an average weighted recall of 0.9201 for 24 runs of their model in their preliminary test set. Additionally, Prellberg and Kramer [2] concluded that all related works have reported good results, but comparisons are not possible because the datasets are rarely publicly available. Mondal et al. [9] reported that their proposed weighted ensemble model, using the kappa values of the ensemble candidates as their weights, has outputted a weighted F_1_-score of 0.886 and a balanced accuracy of 0.862 in their preliminary test set. Among the studies that have used publicly available datasets for ALL, comparisons are not possible because the procedures for evaluating classification accuracy differed among studies. Furthermore, all related studies reported so far have used small datasets. Use of a large dataset is essential for an accurate assessment of state-of-the-art classification technology; we hope the C-NMC dataset can meet this need.

The amount of data in the preliminary test set (1,867 records) is much smaller than the amount of data in the training set (10,661 records), and the image labeling of the preliminary test set data has problems, resulting in lower average accuracy and *η* values the preliminary test set than those of the training set (Table 5). The problem with the image labeling of the preliminary test set data has been explained in the discussion paragraph. Additionally, the F_1_-score of the preliminary test set in the previous studies [1, 2, 9] is 0.84–0.886 and the F_1_-score of the preliminary test set in this study is 0.8894 (obtained by Resnet101-9 ensemble model), indicating that the image labeling of the preliminary test set data needs further confirmation by medical laboratory scientists.

## Conclusions

This Resnet101-9 ensemble model proposed in this study accurately and efficiently classified microscopic images as ALL. The first contribution of this study is the confirmation that an appropriate combination of algorithm hyperparameters for a pre-trained Resnet-101 model can obtain high image classification accuracy. The second contribution of this study is the confirmation that the image classification accuracy of an ensemble model can be enhanced by (1) applying a majority voting strategy and by (2) increasing the number of models (e.g., up to nine) integrated in the ensemble model. Additionally, this study investigated the number of image misclassifications made by the Resnet101-9 ensemble model used to classify ALL in microscopic images. When the Resnet101-9 ensemble model was used to classify ALL in a preliminary test set of microscopic images, accuracy was 85.11%, which was superior to the accuracies obtained by the nine trained Resnet-101 individual models (i.e., Resnet-101-8249(#1), Resnet-101-8184(#2), Resnet-101-8452(#3), Resnet-101-8125(#4), Resnet-101-8061(#5), Resnet-101-8281(#6), Resnet-101-8307(#7), Resnet-101-8002(#8), and Resnet-101-8216(#9) models, accuracy ranging from 80.02% to 84.52%). Other performance measures obtained for the Resnet101-9 ensemble model (i.e., 86.33% precision, 91.71% recall, 72.69% specificity, and 88.94% F_1_-score) were also superior to those obtained by the nine trained Resnet-101 individual models. That is, the Resnet101-9 ensemble model had superior capability in classifying ALL in microscopic images compared to the nine trained Resnet-101 individual models.

## Methods

The research procedure was collecting data and processing microscopic images for classifying ALL that could be used for model training, selecting the pre-trained Resnet-101 model for transfer learning, using Taguchi method to design the combinations of algorithm hyperparameters for the pre-trained Resnet-101 model, fine-tuning and further training the pre-trained Resnet-101 model to classify ALL in microscopic images, comparing and recording classification performance among different trained Resnet-101 models, inferring the best factor-level combination of algorithm hyperparameters, analyzing algorithm hyperparameters in the trained Resnet-101 model for classifying ALL in microscopic images, generating and selecting multiple trained Resnet-101 models for use in a Resnet101 ensemble model, and, finally, comparing the classification performance of the Resnet101 ensemble model with that of trained Resnet-101 individual models. The detailed steps were as follows.

### Collecting data and processing microscopic images for classifying ALL

The microscopic images in the C-NMC dataset were divided into a training set, a preliminary test set, and a final test set. The training set had 10,661 microscopic images, including 7,272 images of ALL (cancer) cells and 3,389 images of Normal cells. The preliminary test set had 1,867 microscopic images, including 1,219 images of ALL cells and 648 images of Normal cells. Since the ground truth for the final test set was not released, the final test set was not used in the study. Image preprocessing by the dataset authors limited each microscopic image to a single cell and a 450 × 450 pixels resolution [8, 10].

To maintain compatibility with the CNN-based architecture and the developed software, each microscopic image was processed as a 224 × 224 × 3 image, where 3 is the number of color channels.

### Selecting the pre-trained Resnet-101 model for transfer learning

The most important characteristics of pre-trained CNN models are network accuracy, speed, and size. The choice of a pre-trained network generally involves a tradeoff among these characteristics. Accuracy in classifying images contained in the ImageNet database [11] is the most common measure of the accuracy of networks trained on the database used in the ImageNet large-scale visual recognition challenge (ILSVRC) [12]. Networks that achieve high accuracy on ImageNet are also expected to achieve high accuracy in other natural image datasets that are used to evaluate performance in transfer learning or feature extraction. The Resnet [13] achieved a 3.57% Top-5 error rate and was the winner of ILSVRC 2015. Therefore, this study selected Resnet-101 (101 layers) for evaluating performance in classifying ALL in microscopic images. Since the Resnet-101 has been trained on more than 1 million images from the ImageNet database used in the ILSVRC, Resnet-101 has learned rich feature representations for a wide range of images and can classify images into 1000 object categories. The image input size for the Resnet-101 is 224 × 224 × 3.

Transfer learning is a machine learning approach in which a model developed for a task is reused as the starting point for a model developed for another task. In transfer learning, a pre-trained CNN model is used to construct a predictive model. Thus, the first step is selecting a pre-trained CNN model from available models. The second step is reusing the pre-trained CNN model, and the third and final step is tuning the pre-trained CNN model for a new task. Depending on the input–output pair data available for the new task, the researcher may consider further modification or refinement of the pre-trained CNN model. Transfer learning is typically much faster in a pre-trained CNN model compared to a CNN model without pre-training.

### Using Taguchi method to design algorithm hyperparameter combinations for the pre-trained Resnet-101 model

The Taguchi method [14–17] is a statistical experimental method of implementing and evaluating improvements in processes and products. The main principle of the method is to enhance quality by minimizing the cause of variations rather than by eliminating them. The Taguchi method minimizes the number of experiments needed to study a large number of design variables. An efficient way to study the effects of several control factors simultaneously is to arrange matrix experiments in orthogonal arrays (OAs). The better factor-level combinations are determined by OAs and signal-to-noise ratios (SNRs).

For the pre-trained Resnet-101 model to achieve high accuracy in classifying ALL in microscopic images, selecting appropriate algorithm hyperparameters was essential. The algorithm hyperparameters for the pre-trained Resnet-101 model in this study were Optimizer, MiniBatchSize, MaxEpochs, and InitialLearnRate. To account for nonlinear effects and to minimize the required number of experiments, a three-level *L*_9_(3^4^) OA was used. Therefore, the combinations of algorithm hyperparameters obtained by the three-level *L*_9_(3^4^) OA were used in a pre-trained Resnet-101 model for classifying ALL in microscopic images.

### Fine-tuning and training the pre-trained Resnet-101 model to classify ALL in microscopic images

**Fig. 3:**
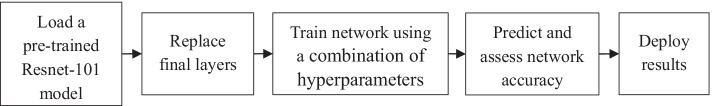
Flowchart of transfer learning procedure used in the Resnet-101 model

To fine-tune a pre-trained Resnet-101 model, transfer learning is often faster and easier than constructing and training a new Resnet-101 model for a new task. Although a pre-trained Resnet-101 model has already learned a rich set of image features, it can be fine-tuned to learn features specific to a new dataset. In this study, the pre-trained Resnet-101 model was fine-tuned to learn features specific to the C-NMC dataset. Since a pre-trained Resnet-101 model can learn to extract a different feature set, the final Resnet-101 model is often more accurate. The starting point for fine tuning deeper layers of a pre-trained Resnet-101 model used for transfer learning is to train the networks with a new C-NMC dataset. Figure 3 is a flowchart of the transfer learning procedure used in the Resnet-101 model.

### Comparing and recording classification performance among different trained Resnet-101 models

The results recorded for the training set and the preliminary test set included (1) accuracy in each run of the experiment, (2) average accuracy in three independent runs, (3) standard deviation in accuracy in three independent runs and (4) *η* value.

Accuracy was defined as the proportion of true positive and true negative results for a population. The concept of SNR was first applied in communications and then in engineering. For engineering applications, a larger SNR (*η*) is preferable and indicates better performance. Taguchi recommended multiplying the common logarithm of SNR by 10, which obtains the SNR in decibels (dB). In this study, the equation for the smaller-the-better characteristic was $\eta \; = \; - 10\;\log \;(\overline{y} - m)^{2}$, where $\overline{y} = \frac{1}{n}\sum\nolimits_{t = 1}^{n} {y_{t} }$(a set of data *y*_1_, *y*_2_, …, *y*_*n*_, accuracy of model training and prediction in each experiment) and *m* = 1 (i.e., the accuracy of the target is 100%).

### Inferring the best factor-level combination of algorithm hyperparameters

A response table was built to find the best factor-level combination of algorithm hyperparameters by using the *L*_9_(3^4^) OA and *η* values. To build the response table, the effects of different factors were set as follows: *E*_*fl*_ = average of sum of *η*_*i*_ for factor *f* at level *l*, where *f* is the factor name, *l* is the level number, and *i* is the experiment number. After the nine experiments for *L*_9_(3^4^) were performed, the response table was used to investigate the *η* of each factor level. The response table showed the average *η* of each factor level and maximum average *η* of each factor. The main objective was to use the response table to find the best level for each factor. The best level was defined as the level with the highest *E*_*fl*_ value in the experimental region. That is, the best factor-level combination of algorithm hyperparameters was inferred according to the results of the nine experiments, even though not all factor-level combinations (i.e., 3^4^ experiments) of algorithm hyperparameters were considered.

### Analyzing the algorithm hyperparameters in the trained Resnet-101 model for classifying ALL in microscopic images

The Taguchi experimental design process uses analysis of variance (ANOVA) to identify important control factors by performing the smallest number of experiments. The ANOVA analyses were performed to find the algorithm hyperparameters in the trained Resnet-101 model that significantly affected the most important characteristic, i.e., accuracy in classifying ALL in microscopic images.

### Generating and selecting multiple trained Resnet-101 models for integration in a Resnet101 ensemble model for classifying ALL in microscopic images

The best factor-level combination of algorithm hyperparameters obtained by the response table for the trained Resnet-101 model was used to classify ALL in microscopic images. The trained Resnet-101 models that had the better performance in classifying ALL in microscopic images were then integrated in a Resnet101 ensemble model used to classify ALL in microscopic images in the preliminary testing data set.

### Comparing the classification performance of the Resnet101 ensemble model with that of trained Resnet-101 individual models

Classification performance was compared in the Resnet101 ensemble model and the trained Resnet-101 individual models. Classification performance was compared in terms of accuracy, precision, recall (i.e., sensitivity), specificity, and F_1_-score values. The five measures were introduced below.

Accuracy is the proportion of true results (both true positive and true negative) in the population. When an information retrieval task is performed, precision is a measure of the relevance of results. Precision is calculated as the positive predictive value (number of true positives over number of true positives plus number of false positives). Another measure of information retrieval performance is recall (sensitivity), which is calculated as true positive rate (number of true positives over the number of true positives plus the number of false negatives). Specificity is calculated as true negative rate (number of true negatives over the number of false positives plus the number of true negatives). The F_1_-score is a function of precision and recall and was used to measure prediction accuracy when classes were very imbalanced. The formula used to calculate F_1_-score in this study was 2 × (precision × recall)/(precision + recall) [18, 19].

## Data Availability

All data obtained and analyzed in this study are included in this article. Microscopic images of acute lymphoblastic leukemia were obtained from the C-NMC dataset available online at https://doi.org/10.7937/tcia.2019.dc64i46r

## Abbreviations

ALL: Acute lymphoblastic leukemia
CNN: Convolutional neural network
ILSVRC: ImageNet large-scale visual recognition challenge
OA: Orthogonal array
SNR: Signal-to-noise ratio
ANOVA: Analysis of variance
SD: Standard deviation
